# Burnout and Online Medical Education: Romanian Students in Lockdown and Their Residency Choices

**DOI:** 10.3390/ijerph19095449

**Published:** 2022-04-29

**Authors:** Ioana Silistraru, Oana Olariu, Anamaria Ciubara, Ștefan Roșca, Ramona Oana Roșca, Silviu Stanciu, Alina Plesea Condratovici, Ioan-Adrian Ciureanu

**Affiliations:** 1 Faculty of Social Sciences and Humanities, Lucian Blaga University of Sibiu, 550024 Sibiu, Romania; ioana.silistraru@ulbsibiu.ro; 2 Faculty of Medicine and Pharmacy, Dunarea de Jos University of Galati, 800008 Galati, Romania; oo105@student.ugal.ro (O.O.); anamaria.ciubara@ugal.ro (A.C.); alina.plesea@ugal.ro (A.P.C.); 3 Department of Chemistry, Physics and Environment, Faculty of Sciences and Environment, Dunarea de Jos University of Galati, 800008 Galati, Romania; oana.gunache@ugal.ro; 4 SAIABA Department-BIOALIMENT Research Center, Faculty of Food Science and Engineering, Dunarea de Jos University of Galati, 800008 Galati, Romania; silvius.stanciu@ugal.ro; 5 Medical Informatics and Biostatistics Department, Faculty of Medicine, Grigore T. Popa University of Medicine and Pharmacy, 700115 Iași, Romania; adrian.ciureanu@umfiasi.ro

**Keywords:** COVID-19, burnout, stress, medical students, medical education, educational burnout, Romania

## Abstract

The primary aim of the study was to investigate the prevalence of burnout in Romanian medical students during the COVID-19 pandemic using the Maslach Burnout Inventory-General Survey for Students (MBI-GS(S)). The presence of burnout was assessed based on Exhaustion (EX), Cynicism (CY) and Professional Efficacy (PE) subscales. The secondary aim of the study was to identify the presence of intentional shift in medical specialty compared to their initial pursued choice within the population investigated. Data was collected online at the end of 2020 and beginning of 2021 through a licensed, customized MBI-GS(S) questionnaire from a sample of 126 Romanian medical students at the two leading medical schools in the country, Iasi (N = 56) and Cluj Napoca (N = 70). Descriptive statistics and bivariate correlations were also applied to describe the data set (age and gender of participants) and the relationship between variables (EX, CY, PE). Subsequently, the MBI-GS(S) group report revealed that 36.5% of the medical students in the sample (46) experienced burnout, with problematic results both in Exhaustion and Cynicism. Exhaustion and Cynicism, which contribute to burnout, showed high scores compared to the average scale (EX = 3.5/2.4; CY = 2.8/1.8), while the Professional Efficacy score was relatively high (PE = 3.8/4.4), showing a protective effect and burnout reduction. One of the main conclusions is that the consequences of burnout in medical students plays a significant role in shaping the future healthcare practitioners’ perception of the medical profession and of patients’ wellbeing. Exhaustion and Cynicism are mainly associated with depersonalization and disconnection from the patient. Another conclusion of the study is that about one third of the respondents (30% Cluj students and 37.5% Iasi students) considered changing residency options. The pandemic has also revealed the limitations of and challenges facing current medical education, and that further research is required to assess the trends in prevalence of burnout in medical students.

## 1. Introduction

Almost two years since the official announcement of the coronavirus pandemic, the sanitary crisis is still far from its conclusion. The impact of COVID-19 on all aspects of life is difficult to estimate. Stress, anxiety, and depression have been identified across professions, but healthcare workers present significant signs of burnout [1,2,3,4]. Recent research suggests that the coronavirus pandemic has hastened the mental health decline of certain professions at the forefront of the virus’s fight, particularly front-line healthcare professionals, but also medical students. Due to the COVID-19 pandemic, most countries have taken severe measures to limit the spread of infection, including in universities. Quarantine and social distancing have already had detrimental effects on people’s mental health, as symptoms of depression, anxiety, and stress have increased dramatically [5].

Most medical schools, due to the COVID-19 pandemic, have required curricular examination and restructuring [6,7,8,9], as well as significant changes in clinical rotations. Studies suggest that the mental health of medical students is significantly under strain, academic stress being the main predictor of burnout. Existing literature corelates educational burnout with emotional exhaustion. Educational challenges during COVID-19 lockdown are generally defined as perceived stress and pressure to face the tasks and student duties [10,11] mostly in an online environment, coupled with an increased workload.

Therefore, the impact of COVID-19 on mental health has led to legitimate concerns about students’ studies and progress during medical education. It was also found in a recent Australian [12] study that emotional exhaustion increased, especially in final year students, who struggled with a lack of clinical experience even before starting to work as resident physicians [13,14,15,16].

The concept of burnout has been used for almost 60 years [17], and it has been constantly developed and refined to evaluate the mental health of professionals and students. MBI has been developed as a multi-faceted research instrument, rather than an individual assessment tool [18] considered as the “gold standard” for assessing burnout [19]. The MBI-GS(S) provides group assessment on three subscales: Exhaustion (example scale item “I feel emotionally drained by my studies”), Cynicism, as loss of enthusiasm and passion for their studies (example scale item “I doubt the significance of my studies”) and Professional Efficacy, as feeling a low level of competence and achievement in academic areas (example scale item “In my opinion, I am a good student”).

According to previous studies [1,3,4,10,20], medical students suffer significant levels of stress throughout college, which is linked to the state of burnout. The Dyrbye study [21] conducted in seven American colleges revealed that 52.8% of medical students had burnout, which was associated with self-reported unprofessional conduct and less altruistic professional values.

Thus, with the COVID-19 pandemic and the profound shifts in private, professional, and social dynamics, burnout and stress in academia are receiving increased attention [22,23]. We aim to investigate the prevalence of burnout in Romanian medical students during COVID-19 pandemic using Maslach Burnout Inventory–General Survey for Students (MBI-GS(S)) on the Exhaustion (EX), Cynicism (CY) and Professional Efficacy (PE) subscales and the presence of intentional shift of residency choice. Thus, looking at how medical students navigate their education and how educational burnout is shaping the future of healthcare workers is of particular relevance [24,25,26,27].

## 2. Materials and Method

This cross-sectional study was conducted using an MBI-GS(S) customized online questionnaire to assess group prevalence of burnout in medical students from two major Romanian medical schools. The questionnaire was administered to a sample of 126 students, based on their availability, as the study was voluntary and anonymous. The representation of Romanian faculties of medicine consisted of Iasi (N = 56) and Cluj Napoca (N = 70). The customized questionnaire was used based on the permission letter obtained on 31 December 2020, by Oana Olariu, through Mind Garden. We compared the average population score in the licensed MBI Group Report with our sample scores regarding the burnout subscales Emotional Exhaustion, Cynicism and Professional Efficacy. We administered the licensed questionnaire in the original English language directly on the Mind Garden platform, as we determined that using English caused no particular difficulty or socio-cultural impediment for our sample group. Data collection was performed through a one-time available licensed questionnaire link, to ensure no duplicate answers were possible. The link to the questionnaire was sent through students’ representatives, providing the informed consent, informing respondents on the purpose of data collection and ensuring full anonymity and voluntary participation.

At the time the customized questionnaire was administered, the students had been taking online classes for almost a year (December 2020–June 2021).

The questionnaire included demographic questions such as age, gender, bachelor’s degree (BA), choice of medical specialty before the pandemic, intention of changing the specialty or profession altogether upon graduation.

The standard questionnaire was customized with additional items and open-ended questions to collect the relevant data concerning the intention to change the desired field of residency upon completion of all six years of medical school. The customized items of the questionnaire were placed after the items in the original instrument, to preserve the psychometric qualities of the instrument.

We used the MBI-GS(S) instrument to measure how frequently the students report Exhaustion, Cynicism and the perception of their level of Professional Efficacy during their studies in the pandemic context. As burnout is a severe problem affecting both students and healthcare workers, its prevalence and severity needs to be evaluated starting with academic studies. In this context, burnout is defined based on the subscales of Exhaustion, whereas students feel overwhelmed, stressed (example scale item “I feel emotionally drained by my studies”); Cynicism, as loss of enthusiasm and passion for their studies (example scale item “I doubt the significance of my studies”); Professional Efficacy, as feeling low level of competence and achievement in academic areas (example scale item “In my opinion, I am a good student”). The groups’ frequency average scores were measured on a Likert scale with responses structured as follows: 0—never; 1—a few times a year or less; 2—once a month or less; 3—a few times a month; 4—once a week; 5—a few times a week; 6—everyday in relation to Exhaustion, Cynicism and Professional Efficacy items.

The statistical analysis was performed with IBM SPSS Statistics v.26 for descriptive statistics and bivariate correlations. 

## 3. Results

Among all of the students (126), in the Cluj sample there were 32.9% (23) male students and 67.1% (47) female students, whereas in the Iasi sample there were 14.3% (8) male students and 85.7% (48) female students. For all students (126) reporting a BA degree, in the Cluj sample (70) there were 31.4% (22) in Mathematics, 47.1% (33) in Natural Sciences, 14.3% (10) in Philology and 7.1% (5) in Social Sciences, while in the Iasi sample (56) there were 48.2% (27) in Mathematics, 35.7% (20) in Natural Sciences, 8.9% (5) in Philology and 7.1% (4) in Social Sciences. Descriptive statistics are presented in Table 1. Our study suggests that 36.5% of the medical students from Cluj and Iasi (N = 126) are currently experiencing burnout. Within the burnout profiles established through MBI-GS(S) scoring, 19 were Engaged, 22 Ineffective, 30 Overextended, 9 Disengaged and 46 Experiencing Burnout.

Only 15.07% (N = 9) of the students with the Engaged profile scored well on all three scales, low on Exhaustion and Cynicism, and high on Professional Efficiency. The profile of Ineffective was scored by 17.46% (22) students, 23.8% (30) were Overextended and 7.14% (9) met the Disengaged profile.

The split subscales of EX, CY and PE scored as follows: for Cluj students, the Cronbach reliability score was α= 0.935 (EX), α= 0.841 (CY), α= 0.845 (PE), whereas for Iasi students, the Cronbach reliability score was α= 0.921 (EX), α= 0.744 (CY) and α= 0.748 (PE). The Cronbach α shows a high reliability for all subscales, as a measure of high internal consistency of the measured items within the group.

Table 1 presents the demographic factors and data regarding the shift in residency options. Among Cluj medical students, 30% (21) claimed that they will pursue a different residency option from the one they initially desired: 13 students with desired clinical residency (3 male and 10 female students) are prepared to change to surgical residency (1 female student) and 12 students will change their option to other clinical residency programs (3 male and 9 female students). Where the first residency option is surgical, 8 students (six male and 2 female students), the students would now rather pursue a clinical residency now (5 students, 3 male and 2 female students) or a different but still surgical residency (3 male students).

In the second group, 37.5% (21) of the Iasi medical students claimed they will also change their initial residency option as follows: 18 students (one male and 17 female students) will change from clinical to surgical (two female students) and 16 students (one male and 15 female students) to a clinical residency different from the one initially desired. Whereas, if the initial option was for a surgical residency, three Iasi students (one male and 3 female) will pursue a clinical residency upon completion of medical studies.

While changing residency option is not unusual during medical studies [14,15], we aimed to assess whether medical students were keener to do so after migrating to online education during lockdown. A further mixed-methods study could analyze whether there are relevant data to corelate the perceived quality of medical education to the desire to change residency choice.

### 3.1. Burnout Subscales and Item Reliability

The subscales measured within the sample demonstrated a good reliability on the three categories EE, CY and PE, as shown in Table 2. For the Cluj student sample, the Cronbach reliability score for the measured dimension EE was *α* = 0.935, CY was *α* = 0.841 and PE *α* = 0.845. For the Iasi student sample, the Cronbach reliability score for EE was *α* = 0.921, for CY *α* = 0.744, and for PE *α* = 0.748. When considering the sample size and the scores *α* scores above 0.7, it can be concluded that the measured dimensions of burnout have good internal validity.

#### 3.1.1. Emotional Exhaustion

In our sample, the group score on Exhaustion is 3.5 compared to a cut-off group score of 2.4 in the general population, as provided by the licensed MBI Group Report. A 3.5 Exhaustion score is above the 3.4 score defined as “I feel burned out by my studies”, as provided by the MBI average score ranking.

#### 3.1.2. Cynicism and Depersonalization

As the feelings of exhaustion and cynicism (defined as depersonalization) are the two main dimensions contributing to burnout, we emphasize that our sample group average CY score is 2.8 in the licensed MBI Group Report. Compared to the average general population score of 1.8, this suggests a high level of depersonalization coupled with a high score of emotional exhaustion for our sample group. A 2.8 CY score falls above the 2.3 average score defined as “I have become less enthusiastic of my studies” and below the 3.2 average score defined as “I have become more cynical about whether my university work contributes anything”.

#### 3.1.3. Professional Efficacy

We look at the average group score in Professional Efficacy as we find indications in the MBI-GS(S) customized licensed group report that high PE reduces burnout, whereas higher Exhaustion and Cynicism contribute to burnout. In our group sample report, we have a PE average score of 3.8 compared to an average general population score of 4.4, as provided by the licensed MBI group report. With respect to average scores per MBI item, our group’s score falls between the 3.5 PE score defined as “I have accomplished many worthwhile things in my studies” and closer to the 3.9 PE average item score defined as “In my opinion, I am a good student”. We conclude that the satisfactorily high score of Professional Efficacy in our sample could indicate an important burnout reducing factor to be considered.

#### 3.1.4. Gender and Subscale Score Correlation

Although correlation does not imply causality, the *p* values were examined to consider a possible relationship between gender and the MBI-GS(S) subscales score results.

Additionally, the reported values of Professional Efficacy scores suggest that high levels of PE have a protective effect on students’ burnout, regardless of gender, as shown in Table 3.

#### 3.1.5. Age and Subscale Score Correlation

In our sample group, the age correlation shows that the protective burnout effect of self-reported Professional Efficacy is higher, while the Emotional Exhaustion and Cynicism dimensions decrease with age, as shown in Table 4.

### 3.2. Group Standard Deviation Results

The groups’ standard deviation values measured for EX, CY and PE show a higher value than the cutoff values offered by the standardized MBI-GS(S) group values, as shown in Table 5. The licensed group report includes a standard deviation value from the general population of 19,000+ people, of diverse segmentation, for comparison. The mean values for the general population show a reasonable agreement among the group members, with values as follows: EX = 1.5, CY = 1.4, PE = 1.1; the smaller the standard deviation within the group, the higher the agreement among the group members. Therefore, our sample scores show a good agreement among the group members on all three subscales, considering the sample size: EX = 1.7, CY = 1.6, PE = 1.3.

## 4. Discussion

Previously, the prevalence of burnout in medical students in Romania was contextualized mostly to the pandemic situation [28,29,30,31,32]. Burnout related to virtual education and online studying has not been fully assessed, but recent studies suggest that research on this topic is growing apace [13,20,33,34,35]. The results of the recent literature suggest that medical students experience burnout during their studies and that the coronavirus pandemic has a significant impact on their well-being. According to existing research [12,36], the costs of burnout are not only professional or academic, but also personal—illness, hopelessness, irritability, impatience, poor interpersonal relationships with family members and other students, or substance abuse.

The COVID-19 pandemic, according to all current estimates, will continue and will develop new facets in affecting the mental status of the general population, health workers, and medical students [10,13,15]. Medical education, like many other areas that involve direct engagement with the beneficiary, or patient, will have to adapt to the new circumstances. High levels of perceived stress and burnout are unavoidable during the pandemic period, and online education can help to fuel them. The data show not only that students’ mental health suffers, but also that on the dimensions of burnout, particularly depersonalization (which is associated with a lack of clinical experience), we estimate that students are affected by concerns about future patient interaction. Recent studies discuss the challenges posed to medical education in lockdown, as the presence of the SARS-CoV-2 virus creates a unique situation where the transitioning to the new medical training format challenges the mental health of students and their wellbeing [20,21,37]. Therefore, as the literature suggests, addressing the opportunity of digital education in medicine, teaching, and clinical practice will be an ongoing challenge for medical institutions. A longitudinal study could provide more information on burnout scores in the student population, monitored throughout the entire period of study. However, the pandemic situation varies, as a recent Croatian study suggests [38], and the first pandemic lockdown and the shift to online learning had no impact on the students’ burnout levels or on their perception over the study program, although our study shows that burnout has affected 36.5% of the respondents. The differences in burnout scores could be related to the accuracy of self-reported data and the specific time in the evolution of the pandemic, as well as to reliable and timely communication.

Additionally, a cross-sectional study from June 2020 on a sample of 165 students from the School of Medicine at University College Dublin, Ireland showed that 54.5% reported stress levels ranging from moderate to high. Higher stress levels were significantly associated with the female gender and international status. There was also a significant association between reported stress and the transition to online education and online knowledge assessment, concerns for personal health, and family health [20].

Another recent study conducted on a sample of 189 students, who filled in a questionnaire before and during the COVID-19 pandemic, showed that the general prevalence of burnout did not differ significantly between the two periods (pre-COVID-19 18.1% compared to COVID-19 18.2%) [12]. However, the prevalence of burnout decreased significantly in students in the fourth year of study (pre-COVID 19 40.7% compared to COVID-19 16.7%), while it increased significantly in students in the final year (pre-COVID-19 27.6% vs. COVID-19 50%). Thus, the researchers found that emotional exhaustion decreased significantly in the fourth year of medical studies, but increased in the final year, whereas cynicism increased in all years. The study suggests that the deterioration of mental health since the onset of the COVID-19 pandemic was reported by 68% of medical students. The main negative effects were related to social contact, study, and stress levels.

Our findings show that female medical students are more vulnerable to emotional exhaustion than male students [39], as the literature already indicates that female students experience higher levels of stress during their education than male students [20,39].

As for the age and burnout prevalence in medical students, studies suggest that although matriculants start with a better mental health status compared to their same-age university student peers, the burnout and depression prevalence is higher in medical students than in college graduates pursuing different careers [40,41,42,43]. As the literature suggests, medical students experience higher levels of mental distress than their age-matched peers and the general population [33]. Studies show that although medical students do not significantly differ from their peers upon starting their graduate courses, as they advance in their medical curricula, the mental health status becomes increasingly deteriorated, and they experience higher levels of study dissatisfaction. The available data comes from a 2012 study [4] at six US medical schools suggesting that although students report a better quality of life, this decreases compared to their peers outside medical studies. However, our sample shows decreasing scores in Emotional Exhaustion and Cynicism for older students, which suggests that although we can compare medical students’ burnout to their peers outside medical education, we should look at how the scores move asynchronously on the age variable. Emotional Exhaustion strongly correlates with Cynicism, involving depersonalization and disconnection with the purpose of medical studies in our sample. The negative PE and CY and PE and EX correlations suggest the protective effect of efficacy, as the higher the self-perceived satisfaction in educational activities, the lower the emotional exhaustion and depersonalization. Therefore, we argue that older students have better efficacy and coping mechanisms to protect from depersonalization and burnout. Longitudinal studies are needed to follow the evolution of burnout in medical student throughout the medical education. 

Several studies have used the MBI-GS and a smaller number have used the MBI-GS(S) questionnaire to assess levels of burnout in students [34,35], especially for medical education during the pandemic, as the three subscale scores help assess the burnout state in students, as well as the protective role of perceived the professional efficacy. Findings related to our study on Romanian medical students provide the research grounds for further studies. A mixed-methods quantitative–qualitative approach could also clarify the causality of variable correlation and identify coping mechanisms and counseling approach to students’ burnout. The existing literature suggests that burnout in students is more accurately identified through qualitative data, whereas the thematic approach suggests coping and building resilience strategies for future healthcare professionals [2,44,45,46].

Burnout in medical students has become more prevalent during the pandemic crisis. A growing body of research seeks to identify the most effective paths of interventions to reduce and subsequently to prevent burnout in students [10,47,48,49].

Existing research suggests that burnout has a significant impact on how students view their choice of career [40]. A longitudinal study from 2006–2007 argues that burnout in medical students predicts the intention of dropping out of school [50], whereas a different cross-sectional study suggests that medical students are more prone in choosing a less demanding specialty to engage in a more manageable lifestyle as future healthcare practitioners [51]. While it remains unclear how students choose their specialty, as a Canadian study suggests [41], personal and professional factors may be the key to choosing a specialty, which leads to a more satisfying career and better care for their patients, as burnout correlates with lower levels of empathy, disengagement and career regret [42]. However, addressing burnout at all stages of medical training, either in medical school or residency is of critical importance for the quality of healthcare and the students’ and residents’ wellbeing [22,42,52,53,54]. In terms of the presence of intentional shift in residency choice, one recent study [55] showed that in the USA, about one-fifth of surveyed medical students were preoccupied with changing their residency choice. From a sample of 1669 American students, 337 (20.2%) respondents believed that the COVID-19 pandemic would affect their residency option, with many being concerned that options were more limited, as well as their access to referees’ letters [55]. In our study, around one-third of the respondents appeared to be considering changing their initial residency option, which might indicate a similar impact of the pandemic, with clinical medical learning affecting Romanian students’ career perception.

## 5. Conclusions

This study, conducted on medical students from two medical schools in Romania, suggests that almost four in ten students experienced burnout during the coronavirus pandemic. Exploring the perceived burnout status is relevant for all medical schools in Romania. In addition, we see an intentional shift in residency choice, although a cross-sectional analysis provides insufficient information on the process. The presence of burnout in medical students, induced by the COVID-19 pandemic, as well as a shift in career alternatives, is highlighted in our study.

Furthermore, the COVID-19 pandemic has already proven that we face limitations in medical education and resources. The assessment of mental health risk should begin during medical school. Identifying the most appropriate lines of intervention for medical students’ mental health is critical, and there is the evidence that positive relationships and psychological interventions can significantly contribute to improving it, as suggested by recent research. More analysis and more comprehensive studies are needed to identify trends and variations in factors that affect medical students’ well-being, but appropriate interventions will contribute to improving it.

The complexity of the issues raised by virtual learning and by the pandemic highlights the importance of conducting comprehensive quantitative and qualitative studies on university students. It is of the utmost importance to accurately determine the factors involved in educational burnout in medical students, as well as to systematize the most effective ways of assisting vulnerable students. Following up on the current research on burnout will allow universities to build a collection of burnout best practices and mental health interventions.

## 6. Limitations

This study has several limitations. One limitation might arise from the cross-sectional design employed. While it offers a view on the current status of medical students, the data collection occurred at a specific point in time. While the findings prove to be very useful to design necessary interventions and adjustment to medical education in Romania during COVID-19 pandemic, the prevalence of burnout in medical students should be followed-up and researched using additional dependent variables, such as years spent in medical school and beyond. Thus, students’ burnout could not be assessed over a longer period of time to observe whether there are changes in scores. Another limitation is related to the absence of comprehensive individual personal information, which could explain an initial more vulnerable mental state of the participants. Finally, another limitation is the self-reporting of certain data, as the questionnaire is administered online. The data collected from our respondents suggest a group burnout status, but individual data is also desirable to formulate indications for adequate interventions.

## Figures and Tables

**Table 1:** Descriptive statistics for participant demographics and relationship to “desired residency” variable.

N = 126	Cluj (N = 70)	Iasi (N = 56)	*p*-Value
Mean age	25.01 ± 4.40	24.69 ± 2.58	0.633
Gender (M/F)	23/47 (32.9%/67.1%)	8/48 (14.3%/85.7%)	0.016
BA Degree			
Mathematics	22 (31.4%)	27 (48.2%)	0.672
Natural sciences	33 (47.1%)	20 (35.7%)	
Social sciences	5 (7.1%)	4 (7.1%)	
Philology	10 (14.3%)	5 (8.9%)	
Desired field of residency before pandemic			
Clinical	52 (74.3%)	45 (80.4%)	0.425
Surgical	18 (25.7%)	11 (19.6%)	
Desired field of residency after 1 year of pandemic studies			
Clinical	55 (78.6%)	46 (82.1%)	0.621
Surgical	15 (21.4%)	10 (17.9%)	

**Table 2:** Descriptive statistics of burnout subscale scores EE, CY and PE split by sample (Cluj and Iasi medical students).

	Scores				Cronbach’s Alpha
	Mean ± SD	Range	Q25	Q75	
Cluj					
EE	16.57 ± 8.80	0.00–30.00	9.00	25.50	0.935
CY	13.12 ± 8.20	1.00–30.00	6.75	25.80	0.841
PE	23.48 ± 8.20	0.00–36.00	19.00	29.25	0.845
Iasi					
EE	18.21 ± 8.07	0.00–30.00	13.25	25.00	0.921
CY	14.66 ± 7.63	3.00–30.00	9.00	18.75	0.744
PE	21.71 ± 7.07	4.00–36.00	11.40	26.75	0.748

**Table 3:** Gender and subscale score correlation.

		Gender	CY	EX	PE
Gender	Pearson Correlation	1	−0.100	0.136	−0.092
	Sig. (two-tailed)		0.267	0.130	0.307
	N	126	126	126	126
CY	Pearson Correlation	−0.100	1	0.551 **	−0.432 **
	Sig. (two-tailed)	0.267		0.000	0.000
	N	126	126	126	126
EX	Pearson Correlation	0.136	0.551 **	1	−0.410 **
	Sig. (two-tailed)	0.130	0.000		0.000
	N	126	126	126	126
PE	Pearson Correlation	−0.092	−0.432 **	−0.410 **	1
	Sig. (two-tailed)	0.307	0.000	0.000	
	N	126	126	126	126

** Correlation is significant at the 0.01 level (two-tailed).

**Table 4:** Age and subscale score correlation.

		Age	C	EX	PE
Age	Pearson Correlation	1	−0.132	−0.306 **	0.142
	Sig. (two-tailed)		0.141	0.000	0.113
	N	126	126	126	126
C	Pearson Correlation	−0.132	1	0.551 **	−0.432 **
	Sig. (two-tailed)	0.141		0.000	0.000
	N	126	126	126	126
EX	Pearson Correlation	−0.306 **	0.551 **	1	−0.410 **
	Sig. (two-tailed)	0.000	0.000		0.000
	N	126	126	126	126
PE	Pearson Correlation	0.142	−0.432 **	−0.410 **	1
	Sig. (two-tailed)	0.113	0.000	0.000	
	N	126	126	126	126

** Correlation is significant at the 0.01 level (two-tailed).

**Table 5:** Standard deviation of subscale scores.

MBI-GS(S) Subscale Scores	Mean (SD)
Exhaustion	1.7 (1.5)
Cynicism	1.6 (1.4)
Professional Efficacy	1.3 (1.1)

## Data Availability

Data available on request from the corresponding author.
